# Melanoma-Bearing Libechov Minipig (MeLiM): The Unique Swine Model of Hereditary Metastatic Melanoma

**DOI:** 10.3390/genes10110915

**Published:** 2019-11-09

**Authors:** Vratislav Horak, Anna Palanova, Jana Cizkova, Veronika Miltrova, Petr Vodicka, Helena Kupcova Skalnikova

**Affiliations:** Czech Academy of Sciences, Institute of Animal Physiology and Genetics, Laboratory of Applied Proteome Analyses and Research Center PIGMOD, 277 21 Libechov, Czech Republic; horakv@iapg.cas.cz (V.H.); palanova@iapg.cas.cz (A.P.); cizkova@iapg.cas.cz (J.C.); miltrova@iapg.cas.cz (V.M.); vodicka@iapg.cas.cz (P.V.)

**Keywords:** melanoma, mutation, genetics, animal model, swine, MeLiM, progression, spontaneous regression, devitalization

## Abstract

National cancer databases document that melanoma is the most aggressive and deadly cutaneous malignancy with worldwide increasing incidence in the Caucasian population. Around 10% of melanomas occur in families. Several germline mutations were identified that might help to indicate individuals at risk for preventive interventions and early disease detection. More than 50% of sporadic melanomas carry mutations in Ras/Raf/mitogen-activated protein kinase (MAPK/MEK) pathway, which may represent aims of novel targeted therapies. Despite advances in targeted therapies and immunotherapies, the outcomes in metastatic tumor are still unsatisfactory. Here, we review animal models that help our understanding of melanoma development and treatment, including non-vertebrate, mouse, swine, and other mammal models, with an emphasis on those with spontaneously developing melanoma. Special attention is paid to the melanoma-bearing Libechov minipig (MeLiM). This original swine model of hereditary metastatic melanoma enables studying biological processes underlying melanoma progression, as well as spontaneous regression. Current histological, immunohistochemical, biochemical, genetic, hematological, immunological, and skin microbiome findings in the MeLiM model are summarized, together with development of new therapeutic approaches based on tumor devitalization. The ongoing study of molecular and immunological base of spontaneous regression in MeLiM model has potential to bring new knowledge of clinical importance.

## 1. Introduction

Skin cancer is a heterogeneous group of oncological diseases that demonstrate worldwide increasing incidence and include cutaneous melanoma (also known as malignant melanoma) and non-melanoma skin cancers (with basal cell carcinoma and squamous cell carcinoma being the most frequent). Non-melanoma skin cancers are more frequent, affect mainly the elderly population, and demonstrate relatively lower aggressiveness, metastatic activity, and mortality. On the contrary, melanoma represents the least frequent but most aggressive skin cancer resulting in 65% of all skin cancer deaths. Skin damage caused by sunlight (ultraviolet radiation) exposure is the main risk factor for development of such skin malignancies [1,2,3,4].

Melanoma cells arise from neoplastic transformation of melanocytes, which are pigmented cells originating from melanoblasts. Melanoblasts are non-pigmented precursors derived from multipotent neural crest cells, which migrate during embryonic development to the target tissues. Mature pigmented melanocytes are dispersed in the basal layer of the epidermis and in hair follicles, where they are responsible for skin and hair color. Moreover, melanocytes are naturally present in the iris of the eye, inner ear, nervous system, heart, and other organs [5]. The cutaneous melanoma is the most frequent form. Rarely, neoplastic transformation can arise during fetal development, manifesting as neonatal congenital melanoma [6]. More common is postnatal neoplastic transformation, giving rise to several distinct melanoma variants [7]. In affected humans, long-term monitoring of growing skin lesions and their particular biological analyses are not possible for ethical reasons. Thus, various animal models serve as indispensable objects for detailed research of melanoma and development of new therapeutic procedures. Swine represents an invaluable model with anatomical and physiological resemblance and considerably similar skin architecture to human [8,9].

## 2. Human Melanoma

### 2.1. Incidence

The incidence of cutaneous melanoma steadily increased over the last 50 years, particularly in fair-skinned populations in Europe, North America, Australia, and New Zealand [10]. The highest incidence is recorded in Queensland, Australia (approximately 50 cases per 100,000 people per year); in European populations, the incidence reaches 15–20 cases per 100,000 per year [11]. Almost 100,000 new cases are predicted to be diagnosed in 2019 in the United States, making melanoma the fifth most frequently diagnosed cancer [12]. Rising incidence was also reported for young and middle-aged people [10,13]. The increasing incidence is accompanied by increasing mortality from such a disease. However, due to education on melanoma prevention, early diagnosis, and advances in treatment, a descent in mortality is expected in the following years, at least in developed countries.

### 2.2. Risk Factors

The risk of melanoma development depends mainly on interaction between environmental exposure and susceptibility of the host [13]. The major environmental cause of melanoma is sun exposure, particularly intermittent (short and intense) sun exposure and the number of sunburns [14]. Additional environmental factors, such as exposure to cosmic radiation (e.g., in airway pilots and crew), polycyclic aromatic hydrocarbons, benzene, heavy metals, and other chemicals, were suggested to play a part in the etiology of the disease. However, the information from studies of such factors is not strong [14]. 

The most important host risk factors are the number and type of melanocytic nevi. Presence of a high number of nevi, large nevi (diameter over 2 mm), and/or dysplastic or atypical nevi, even on body parts not chronically exposed to sunlight, is associated with an increased risk of melanoma [14]. For example, individuals with more than 100 normal nevi are at almost seven-fold higher risk than people with fewer than 15 nevi [14]. Skin, hair, and eye colors, ability to tan, and propensity to burn are additional host factors influencing melanoma development [13,14]. As approximately 10% of cases occur in families, genetic factors contribute to the susceptibility to melanoma. The discovery of melanoma susceptibility genes and their mutations could lead to development of more accurate prediction and screening tools to identify high-risk populations and to identify new therapeutic targets or prevention strategies [14,15].

### 2.3. Genetic Background in Melanoma

#### 2.3.1. Germline Mutations in Familial Melanoma

In the human population, an increased incidence of melanoma observed in relatives of affected individuals led to the suggestion of a hereditary cause [16]. First genetic studies on melanoma cell lines established from patient metastases identified loss of heterozygosity in several autosomal and X-linked loci [17]. Five years later, deletion within the human chromosome 9p.21 region was identified [18]. The linkage analysis of melanoma prone families from Australia confirmed the existence of a melanoma susceptibility gene in region 9p [19]. Kamb et al. identified a candidate gene in the 9p region as the cyclin-dependent kinase inhibitor 2A (*CDKN2A*) gene, encoding the p16^INK4A^ protein, which is an inhibitor of cyclin-dependent kinase 4 (CDK4). All three identified mutations in the *CDKN2A* gene changed the p16 amino-acid sequence [20]. Many *CDKN2A* gene mutations were later observed in populations of various countries including southern Sweden [21], Massachusetts, United States of America (USA) [22], United Kingdom [23], France [24], and Queensland, Australia, where the mutations were found only in high-risk families [25]. An additional transcript variant of *CDKN2A* gene was discovered in 1995 by Quelle et al., sharing exons 2 and 3 with p16 but having a different exon 1, and was named p19^ARF^ in mouse [26]. The human counterpart (p14^ARF^) was identified three years later [27]. Currently, germline *CDKN2A* mutations are observed in 20–40% of families with hereditary melanoma across continents [28]. More than 60 different mutations in the *CDKN2A* gene were found in hereditary melanoma families, with the majority of them represented by missense mutations in p16 [29]. In contrast, incidence of somatic *CDKN2A* mutations in sporadic melanomas is very low [30].

In 1995, a mutated *CDK4* was found in cultured melanoma cells and metastatic tissue. This mutation prevented binding of p16^INK4A^ to CDK4, thus obstructing inhibition of the CDK4 enzyme activity [31]. A *CDK4* mutation was later found in two unrelated melanoma families [32], and the role of *CDK4* mutations in melanoma development was confirmed [24]. In 17 familial melanoma pedigrees, two germline mutations in *CDK4* were observed by Puntervol et al. [33]. Both *CDKN2A* and *CDK4* represent high-susceptibility genes for malignant melanoma, i.e., mutation in such genes greatly increases the chance of melanoma development.

Additional gene mutations were identified as causal for predisposition to melanoma itself or in combination with other cancers in the last decade. Germline mutations in the breast cancer 1 (BRCA1)-associated protein-1 *(BAP1*) gene were found in highly metastatic uveal melanoma [34] and later also in familial cutaneous melanoma [35,36]. The *BAP1* mutations frequently lead to loss of BAP1 expression (e.g., due to homozygous deletions, premature stop codon, or missense mutations). Loss of *BAP1* expression was observed in 5% of cutaneous melanomas by immunohistochemistry [37]. The BAP1 functions as part of the DNA damage response proteins promoting repair of DNA double-strand breaks [38]. However, the exact mechanism of *BAP1* mutations that promote melanoma genesis is yet to be elucidated [39].

Germline mutation in telomerase reverse transcriptase (*TERT* gene) [40] and other proteins, which protect the ends of chromosomes from deterioration and the cells from senescence, were also reported in melanoma affected families. Mutations in the protection of telomeres 1 (*POT1*) gene may lead to insufficient capping of telomeres by the shelterin complex and may also regulate telomerase function [39]. Loss-of-function, missense mutations or other *POT1* variants were observed in familial melanoma patients in the United Kingdom, the Netherlands, and Australia [41] and in another study also in Italy, USA, and France [42]. Incidence of pathogenic germline mutations of *POT1* is low (~2–5%) [43]. Mutation in additional shelterin complex genes (adrenocortical dysplasia protein homolog, *ACD*; telomeric repeat-binding factor 2-interacting protein 1, *TERF2IP*) were found in familial melanoma patients [44].

Mutations in the microphthalmia (mi) locus in mice are causative for several defects, including small unpigmented eyes and lack of skin melanocytes [45]. A human homolog, microphthalmia-associated transcription factor (*MITF*) gene codes for a transcription factor activating expression of tyrosinase, a rate-limiting enzyme in melanin biosynthesis [46]. MITF is also a major transcriptional regulator of melanoma inhibitor of apoptosis (ML-IAP) expression in melanoma tissues. This suggests that MITF has pro-survival activity in melanoma progression [47]. *MITF* germline mutations increase risk of cutaneous melanoma development by three- to five-fold [39]. *MITF* amplification is more prevalent in metastatic disease and correlated with decreased patient survival [48]. Mutations in the *MITF* gene are found not only in melanomas but also in other cancers, such as renal cell carcinoma [49]. 

As mutations in high-susceptibility genes greatly increase risk of melanoma development, individuals carrying *CDKN2A*, *CDK4*, *BAP1*, *POT1*, or *MITF* mutations should be educated on the importance of melanoma prevention and early detection and should undergo regular medical skin examination [15]. Unfortunately, it still remains uncertain how these mutations influence patient phenotypes, as the melanoma risk is influenced by variations in penetrance, environmental exposure, and coinheritance with low-susceptibility genes [29,39].

Low-susceptibility genes are genes with variants increasing risk of melanoma development with lower penetrance. Melanocortin 1 receptor (*MC1R*) gene variants are associated with red hair and fair skin, a skin phototype with higher risk of melanoma development [50,51]. Presence of *MC1R* variants, together with *CDKN2A* mutations, significantly increases melanoma risk [52]. 

The protective role of calcitriol, a hormonal derivate of vitamin D3, was confirmed in melanoma studies [53,54]. Several polymorphisms of the vitamin D receptor (*VDR*) gene have a supporting effect in melanoma formation and correlate with a negative outcome in affected patients [55].

Epidermal growth factor (EGF) is relevant to wound healing, proliferation of epidermal tissues, and tumorigenesis. Functional polymorphisms of this gene are associated with melanoma development [56]. 

Many other gene variants may increase melanoma risk. Due to the only partial penetration and combination with other (host, environmental) factors, low-susceptibility genes are difficult to identify. More detailed information and additional gene candidates can be found in several reviews [14,39,57,58,59,60]. Genome-wide association studies (GWAS) are used to investigate the entire genomes for single-nucleotide polymorphisms or other gene variants associated with diseases. GWAS allow examination of genes previously not known to be connected to a disease, especially in polygenic diseases with incomplete penetrance, such as many cancers. Results from 11 GWAS in melanoma identified more than 20 loci, including skin pigmentation, epidermal development, telomere maintenance, and cell-cycle progression gene loci, to be associated with melanoma [61]. Pigmentation-related genes, such as *MC1R* (discussed above), oculocutaneous albinism type 2 (*OCA2*), Agouti signaling protein (*ASIP*), tyrosinase (*TYR*), Tyrosinase-related protein 1 (*TYRP1*), solute carrier family 45 member 2 (*SLC45A2*), and a locus encoding interferon regulatory factor 4 (*IRF4*) and exocyst complex component 2 (*EXOC2*), associate with increased risk of melanoma and also other cutaneous malignancies (basal cell carcinoma, squamous cell carcinoma) [62,63].

#### 2.3.2. Somatic Mutations in Sporadic Melanoma

**Figure 1:**
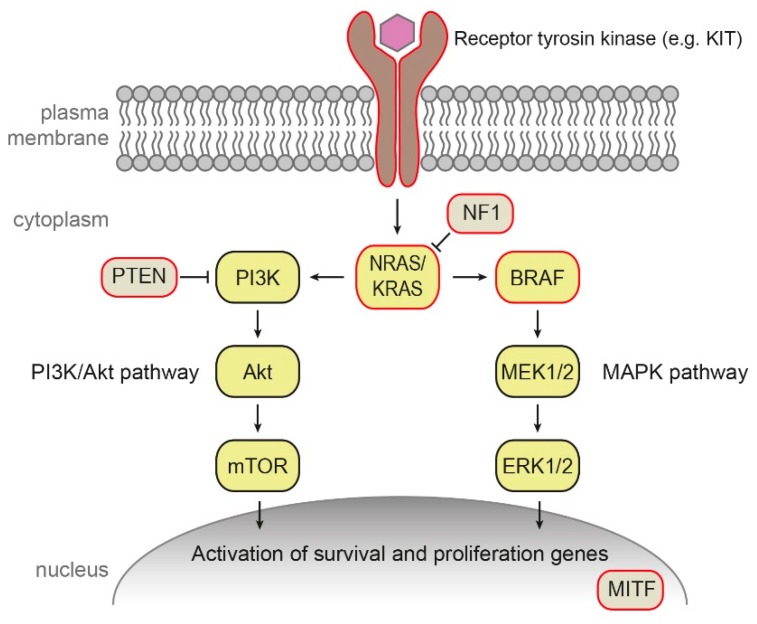
Mitogen-activated protein kinase (MAPK/MEK) and phosphatidylinositol 3-kinase (PI3K)/protein kinase B (Akt) pathways involved in sporadic melanoma. Mutations frequently present in melanoma tissue are highlighted in red.

The majority (~90%) of cutaneous melanoma cases occur sporadically without any records in family pedigree. Such tumors result from somatic mutations and other changes accumulated in the pigmented tissues during the life of an individual. In the majority of sporadic human melanomas, mutations activating the mitogen-activated protein kinase (MAPK/MEK) pathway (Figure 1) are present, affecting mainly *BRAF*, *NRAS*, or neurofibromin 1 (*NF1*) genes (see below).

*BRAF* encodes B-Raf signal transduction serine–threonine kinase regulated by Ras and activating the MAPK signaling cascade [64]. About 50% of cutaneous melanomas carry a mutation in *BRAF* gene, which is in approximately 50% cases represented by V600E substitution, followed by V600K (10–15%) and several less frequent mutations [65]. Interestingly, mutation *BRAF* V600E was detected also in a majority of benign nevi [65]. 

The Ras proteins are essential regulators the MAPK and the phosphatidylinositol 3-kinase (PI3K) pathways [66]. In 10–15% of melanomas, mutations in *NRAS* occur, predominantly in codon 61. Such *NRAS* mutations are an adverse prognostic factor [67]. Mutations in *KRAS* are rare in cutaneous melanoma (2% of cases), in contrast to other cancers such as colorectal cancer [67]. Interestingly, *KRAS* mutations were detected in several mouse melanoma models and melanoma cell lines [67].

Neurofibromin 1 is a negative regulator of Ras. NF1 inactivation leads to the constitutive activation of the MAPK and PI3K pathways. Mutations inactivating NF1 were reported in approximately 50% of melanomas [66].

Increased expression of receptor tyrosine protein kinase erbB-3, also known as human epidermal growth factor receptor 3 (HER3), a member of the EGFR family of receptor tyrosine kinases, was described as a marker of poor prognosis in melanoma [65]. Less than 2% of cutaneous melanomas carry mutation in transmembrane receptor tyrosine kinase *KIT* [11,65].

Amplifications of the *MITF* gene were observed in 20% of metastatic melanomas and are associated with decreased five-year survival. It was suggested that MITF can be activated by the MAPK pathway in malignant melanoma development [66]. 

Mutation in other molecules and pathways outside of the MAPK pathway were also reported in sporadic melanoma, e.g., mutations and deletions in phosphatase and tensin homolog (*PTEN*), which encodes a phosphatase and a key regulator of the PI3K signaling pathway, as well as mutations in p53, telomerase catalytic subunit *TERT*, cell-cycle regulating proteins, and many others [65,66,68]. 

According to the most prevalent significantly mutated genes, The Cancer Genome Atlas Network recently provided a schema for cutaneous melanoma genomic classification into four subtypes: mutant *BRAF*, mutant *RAS*, mutant *NF1*, and triple-WT (wild-type) [69]. Elucidation of important mutations in melanoma led in the last decade to the development of targeted therapies that improved survival of melanoma and also other cancer patients. The examples include B-Raf inhibitors that are used in B-Raf V600E and V600K mutated cancers or MEK inhibitors for treatment cancers with activated upper parts of the MAPK cascade [64]. The genetic classification of melanoma represents a significant step toward personalized medicine from both prognostic and treatment points of view [70].

### 2.4. Regression

Spontaneous regression is a disappearance of the tumor or its part in the absence of any treatment. It occurs more frequently in melanoma than in other human tumors [71]. However, this observation might be biased by easier identification and visualization of cutaneous tumor regression compared to internal cancers such as breast cancer and colon cancer [72]. Signs of depigmentation can develop in local parts of the melanoma lesions. Such partial regression is observed in about 20% of primary human melanomas. The complete melanoma regression is a very rare phenomenon with only 57 described cases in years 1866–2009 [73] or 52 well-documented cases in the literature between 1963 and 2014 [74].

Spontaneous regression is probably related to high immunogenicity of the malignant melanoma, which is able to attract infiltrating immune cells into the tissue. These cells then destroy the tumor and create an inflammatory environment that further activates the immune system [75,76]. The possible triggers of regression may include trauma (including surgery), infection, or immune response of the patient [73,77]. Histopathologically, the early regression involves inflammatory changes with lymphocytic infiltration, as well as the presence of melanophages [73]. Later, dense fibrotic tissue is formed with few or no lymphocytes, and the tissue changes are similar to those observed in a scar [78]. 

Opinions on the prognostic significance of spontaneous regression remained controversial for years. On the base of current clinical and histological data, the regression of melanoma seems to be a positive prognostic factor associated with a lower possibility of metastases in sentinel lymph nodes [78,79].

### 2.5. Therapy of Melanoma

Current melanoma therapies rely mainly on surgical excision, chemotherapy, targeted therapy, and immunotherapy. Tumors in situ are treated by surgical excision, which is highly effective for early cancer stages and patients with early diagnosed melanoma (stage 1A or 1B), showing a 10-year survival rate of 94–98% [80]. Surgery may be combined with lymphadenectomy in patients with positive findings in sentinel lymph node biopsy. In specific cases, the surgery may be combined with radiotherapy [70]. Metastatic disease is mostly inaccessible by surgery. Chemotherapy is used in selected late-stage melanoma patients with progressive or relapsed disease [81].

The identification of mutations in the B-Raf kinase constitutively activating the MAPK pathway triggered new targeted therapies with small-molecule inhibitors of B-Raf and/or MEK kinases. These inhibitors initially showed an excellent response with a significant reduction of tumor burden. Unfortunately, MAPK inhibitors frequently face the development of drug resistance within months of application [81,82].

As melanoma is a highly immunogenic tumor, attempts to boost the patient’s immune system against the tumor by immunotherapy or vaccines are applied in advanced melanoma stages. Since 1998, interleukin-2 (IL-2) was approved for such a purpose, followed by interferon α-2b (IFNα-2b) in 2011 [81]. Current immunotherapies are aimed at increasing cytotoxic cluster of differentiation 8 (CD8)^+^ cell number or efficacy, mostly by targeting cytotoxic T-lymphocyte-associated antigen 4 (CTLA-4) and the programmed cell death protein 1 (PD-1)/programmed death-ligand 1 (PDL-1) pathways [70]. Development of such drugs, called immune checkpoint inhibitors, marks a major progress in treatment of several solid tumors including metastatic melanoma [81]. Additional immune checkpoint inhibitors targeting new molecules are in clinical trials [83,84,85]. Numerous clinical trials are also ongoing to explore efficacy, safety, and tolerability of immunotherapies in combination with chemotherapy, MAPK pathway inhibition, oncolytic viruses, gut microbiota modulation, and other approaches [83,84,86].

## 3. Animal Models

Direct melanoma research in affected humans is not possible for procedural and ethical reasons. Therefore, various animal models were developed that allow detailed study of cancer development, growth, and metastasis, as well as potential therapy of this life-threatening disease. Selected animal models are introduced in the sections below with an emphasis on those with spontaneously developing melanoma.

### 3.1. Non-Mammalian Models 

Non-mammalian species, particularly *Drosophila melanogaster* and *Danio rerio*, are popular to study the development of various diseases including cancer, mainly because of easy breeding, short generation interval, and the possibility of genetic modification, allowing cell transplantation experiments and drug screening [87,88,89,90]. Optical transparency of certain models/developmental stages is advantageous for in vivo imaging [90].

Non-vertebrate species such as fruit fly (*Drosophila melanogaster*) are particularly useful for the study of gene and pathway regulations associated with tumor development or progression [91]. Current transgenic tools allow knockdown or overexpression of any fruit-fly gene in almost any tissue at any stage of development or adulthood [90]. In melanoma, fruit fly was used to study the effect of *Tum1* (tumorous-lethal) mutation on melanotic neoplasm growth [92].

Central American fish *Xiphophorus* was historically among the first fishes in cancerogenesis studies, as, in this fish, various cancers, including melanoma, spontaneously evolve in nature. In 1928, monitoring of *Xiphophorus* offspring led to the discovery of hereditary melanoma transmitted by Mendelian genetics. Such experiments laid a base for existence of cancer-causing genes, currently called “oncogenes” [88,93]. In *Xiphophorus,* melanoma can be also induced by various physical and chemical means, such as ultraviolet (UV) radiation [94,95], X-rays, *N*-methyl-*N*-nitrosourea, or *N*-ethyl-*N*-nitrosourea [93]. A *Xiphophorus* gene associated with aggressive melanoma formation was identified as Xiphophorus melanoma receptor tyrosine-protein kinase (*Xmrk*). The *Xmrk* gene encodes a membrane tyrosine kinase, which has homology to the epidermal growth factor receptor (*HER* gene) [96].

Zebrafish (*Danio rerio*) was the first fish species used to study chemical cancerogenesis [87]. Availability of genetic manipulation enabled generation of transgenic zebrafish models. Patton et al. generated transgenic zebrafish expressing common V600E mutant *BRAF* under the control of the *MITF* promotor. In p53-deficient fish, activated B-Raf induced development of invasive melanomas [97]. Since that time, many transgenic zebrafish models were created for oncogenesis studies [98,99,100,101,102]. Transplantation experiments revealed that human melanoma cells grafted to zebrafish kept their phenotype, i.e., proliferated, migrated, stimulated angiogenesis, and produced melanin [103]. Transplantation of the ZMEL1 melanoma cell line derived from a transgenic zebrafish into transparent zebrafish strain reliably gives rise to widespread metastases [104].

Medaka (*Oryzias latipes*) represents an additional fish model for melanoma studies. Medaka is easy to breed, produces externally developing transparent embryos, does not have naturally occurring tumors, and transgenic technologies are available to modify its genome. Transgenic medaka was developed to express the *Xmrk* gene under the control of a pigment cell-specific promoter. Several stable transgenic medaka lines with spontaneously developing melanomas at 100% penetrance were created [105]. The transcriptomic comparison of medaka and human melanoma revealed molecular conservation between fish models and human tumors at various levels, including the expression of classical melanoma markers, upregulation of N-cadherin, downregulation of E-cadherin, inhibitors of cell-cycle, growth-promoting genes, and inhibitors of apoptosis [106].

### 3.2. Mammalian Models

#### 3.2.1. Mouse Models

The first mouse melanoma models were created by the subcutaneous application of melanoma cells [107] or chemical induction [108]. Later, for study of genetically determined melanoma, the transgenic mice were developed by integration of a recombinant gene comprising the tyrosinase promoter and the simian virus 40 early (SV40E) region. Affected animals developed ocular and cutaneous melanomas, which were histopathologically similar to the human ones [109]. These Tyr-SV40E mice were used in a donor–acceptor study, where grafts of full-thickness skin from a high-susceptibility line were transplanted to the host of a low-susceptibility line (of the same inbred strain). Pigment cells persisted as expected; however, at the outermost rim of all the grafts, a blackened edge arose. Later, the hyperpigmentation spread to surrounding skin, and one or more cases of local thickening arose, signaling vertical growth. These tissue areas became early melanomas. It was noteworthy that all melanomas were strictly confined within the grafts. The origin of melanomas from the host grafts was confirmed by Southern blot analysis of DNA [110]. These results indicate that mouse is a useful model for both allograft and xenograft studies [111].

In 1996, spontaneous melanoma formation was observed as a side effect of the construction of a transgenic mouse strain. Such a result showed how uncontrollable the insertion of genetic information can be, affecting areas other than originally intended [112]. Affected animals from this study were used for establishment of a transgenic melanoma-bearing mouse line that allows the detailed study of development and spreading of melanoma lesions in mice [113].

Current melanoma research relies mostly on syngeneic, xenograft, and genetically engineered models. In syngeneic models, mouse melanoma cells are inoculated into inbred animals of the same genetic background. Due to the presence of a fully functional immune system, syngeneic models allow the investigation of melanoma behavior, metastases formation [114], and immune cell role in tumor microenvironment or cancer immunotherapies [115]. The most commonly used model is B16 melanoma cell grafting to C57BL/6 mice [116].

Severe combined immunodeficiency (SCID) mice became one of the most popular animal models of many human diseases including cancer due to the possibility of inoculating different cell lines and even xenografts without rejection. Patient tumor-derived xenografts (PDX) into immunocompromised mice are widely used to study the response to therapeutic agents [117] or metastasis formation [116]. However, PDX mice lack a functional immune system, which hampers the investigation of immunotherapies. Thus, mouse PDX models with partially or completely humanized immune systems were recently developed. The human immune system can be introduced to irradiated or immunodeficient mice by grafting of purified human CD34^+^ hematopoietic stem cells [118]. 

Genetically engineered mouse models are extensively used to study the effects of genetic alterations in melanoma initiation, progression, and metastasis, as well as for drug efficacy assessment [116]. Transgenic models were the subject of several recent reviews, where detailed information can be found [116,119,120]. The presence of germline mutations in genetically engineered mouse models may affect developmental and reproductive fitness, as well as lead to the formation of tumors in other tissues [119]. Inducible or tissue-specific gene expression may help to overcome such limitations. For that purpose, RCAS/TVA mouse models were developed. Such systems use an RCAS viral vector, derived from the avian sarcoma-leukosis virus, which can deliver genes up to 3 kb. Mammalian cells to be affected by this vector must be engineered to express receptors allowing avian virus entry (TVA) on their surface, e.g., transgenic mice expressing TVA early in melanocyte development from the tyrosinase-related protein 2 (TRP2) promoter [119]. The RCAS/TVA model allows investigation of the carcinogenic potential of candidate oncogenes in somatic cells in vivo [121]. A different model uses conditional melanocyte-specific expression of *BRAF* V600E mutation combined with conditional *PTEN* tumor suppressor gene silencing under the control of Cre recombinase expression from the tyrosinase promoter (BPT-mouse), leading to metastatic melanoma formation with 100% penetrance [122]. The Cre/LoxP system was later used for spatiotemporal control of other oncogene expression in melanoma development [120]. 

Interestingly, the induction of cutaneous melanoma with ultraviolet radiation was not very successful in non-transgenic mice. Therefore, several transgenic mice lines were established that are susceptible to melanoma induction by UV [123,124,125].

Each mouse model system possesses unique advantages and disadvantages [115,116,119]. Moreover, the interpretation of results from mice melanoma models should take into account the different location on melanocytes in skin, which is dermal in mice in contrast to epidermal in human [125,126]. Such a different microenvironment may influence melanoma growth and spreading.

#### 3.2.2. Dog Models

Spontaneously developed pigmented lesions are common in dogs and share some features with human pigmented lesions. In purebred dogs (especially Standard and Miniature Schnauzers, Doberman Pinschers, Scottish Terriers, Irish and Gordon Setters, and Golden Retrievers), the prevalence of this disease is higher, which indicates its genetic basis [127]. Canine dermal melanoma is largely a benign tumor; however, uveal, oral, and mucocutaneous melanomas are aggressive forms frequently metastatic into regional lymph nodes and lungs. They are poorly responsive to conventional therapy [128]. The oral cavity is the most frequent location of canine melanomas (approximately 60% of cases) and such tumors mimic human mucosal melanomas [129]. Results from a study of tumor suppressors in melanoma samples and melanoma cell lines derived from dog tumors indicate that loss of function of certain proteins is a common occurrence that may contribute to the origin of canine melanomas. The most frequent abnormality was significant reduction or loss of p16 protein expression. In the case of p53 tumor suppressor, the exclusion of protein from the nuclear compartment was seen in almost all of the studied samples [130]. Transcriptomic analysis of canine oral melanoma revealed mutations in *NRAS* and *PTEN* genes, but not in *BRAF* [131], as well as upregulation of matrix metalloproteinase 2 (*MMP2*) and downregulation of *MMP7* [132]. Activation of the PI3K/protein kinase B(Akt) pathway was detected in malignant melanomas on distant extremities [133]. In a genomic study of 27 canine malignant melanoma tumors, mutations in genes including *BAP1*, *KIT*, *KRAS*, *NRAS*, *PTEN*, and *TP53* were found, while no mutation in *TERT* promoter, *BRAF*, *CDK4*, *MITF*, or *NF1* genes was detected. In approximately 20% tumors, mutations in *PTPRJ* (protein tyrosine phosphatase, receptor type J), a putative tumor suppressor gene not previously shown to have frequent inactivating point mutations in cancer, was observed [134]. Dog melanomas and their epidemiological, clinical, histological, and genetic comparison to human ones were the subject of a recent excellent review by Prouteau and André, where additional information can be found [129].

#### 3.2.3. Equine Models

Spontaneous occurrence of dermal melanomas was seen in horses with a gray coat color [135]. In Camargue-type gray-skinned horses, multiple melanomas were observed. Most horses had tumor(s) underneath the tail, and less often in the perianal region, on lips, in the eyelids, and in genitals. The skin tumors were rarely seen in other body regions. In some of the strongly affected animals, the metastases developed; however, clinical examination and other observations suggest that melanomas in these horses are clinically different to those in human patients [136]. In graying white horses from the Old Kladruber strain, melanomas usually naturally occur at the age of 5–6 years, and statistically significant differences between the sire lines indicate a possible influence of heritable factors [137]. The 4.6-kb duplication in the intron of the syntaxin 17 (*STX17*) gene was found to cause the graying in horses and is associated with a high incidence of melanoma and vitiligo-like skin depigmentation [138]. Transcription factor MITF is appropriate for the identification of melanocytic cells in horse melanoma. Moreover, the receptor for activated C kinase 1 (RACK1) protein was found as a useful marker to discriminate melanoma cells from healthy skin and melanocytic lesions [139].

## 4. Swine Melanoma Models

Spontaneous occurrence of melanoma in pigs is generally very low. Skin tumors were occasionally observed in pigmented meat breeds such as Duroc, Bazna, and Iberian pig. Metastases into lymph nodes and visceral organs were found in the affected Duroc pigs [140,141,142,143]. An extensive study of 747,014 swine carcasses (without information about breed) revealed 220 cases (i.e., 0.03% only) with cutaneous and lymph node lesions suggestive of melanoma. Histological analysis of samples taken from 176 cutaneous lesions revealed that almost all of them (with the exception of two non-regressing melanomas) were spontaneously regressing [144]. Monitoring offspring from the crossing of Duroc pigs suggested the inherited characteristics of melanocytic tumors [145]. Using selective breeding, three miniature pig models with hereditary melanoma were established: the Sinclair miniature swine, the Munich miniature swine Troll, and the melanoma-bearing Libechov minipig (MeLiM). Melanomas in these three models show similarities such as early postnatal development, histopathology, and spontaneous regression connected with depigmentation.

### 4.1. Sinclair Miniature Swine

The Sinclair miniature swine was derived from the Hormel miniature pig (also known as the Minnesota miniature pig) that was developed as a small pig model at the Hormel Institute (University of Minnesota, Austin, USA). A portion of the original Sinclair herd was moved to the University of Missouri (Columbia, USA) in 1965 and then to the Sinclair Comparative Medicine Research Farm (Columbia, USA). The first Sinclair swine with cutaneous melanoma observed in this strain appeared in 1967 [146]. The incidence of melanoma changed during development of this pig model. The initial incidence of pigmented cutaneous lesions was 21% [146]. In subsequent generations, the incidence was highly influenced by selective breeding, reaching the highest level around 60% in newborn offspring of both affected parents [147,148]. Black pigs showed multiple primary skin lesions of variable size and appearance (exophytic, flat, ulcerated, locally necrotic) that were often present already at birth (congenital) or developed postnatally. On the contrary, no tumors were found in piglets with the red coat color.

Cutaneous pigmented lesions in the Sinclair miniature swine have a variety of histopathologic forms showing many similarities to human lesions. They were classified as benign nevi, superficial spreading melanoma, or nodular melanoma metastatic to lymph nodes and visceral organs (mainly lungs and liver). Skin tumors spontaneously regressed during postnatal life, and this was often accompanied by a local or generalized depigmentation of skin and bristles. Complete regression of melanoma including metastatic regional lymph nodes was also observed [147,149,150,151,152]. The proportion of animals with melanoma regression ranged between 85% and 100%. Detailed histological evaluation of the regressing melanomas revealed a biphasic immunological process. The first phase took place mainly during the second month after birth and was characterized by massive macrophage infiltration. This initial phase displayed tumor mass with less variation and was followed by regrowth of the residual melanoma tissue. The second phase (starting around the beginning of the fourth month of age) showed lymphocyte infiltration and complete elimination of melanomas [153]. Immunophenotyping of tumor-infiltrating lymphocytes in the second regression phase revealed significantly more cytotoxic (CD4^−^/CD8^+^) T-lymphocytes compared to peripheral blood, whereas percentages of the T-helper (CD4^+^/CD8^−^) lymphocytes and double-positive (DP) CD4^+^/CD8^+^ T-lymphocytes were reduced. The percentage of B-lymphocytes (CD1^+^) was very low [154]. These results demonstrate that the cytotoxic T-lymphocytes play the main role in the final elimination of melanoma cells during the second regression phase. However, role of specific antibodies in the spontaneous regression cannot be excluded, as antibodies against melanoma antigens were found in sera collected from the Sinclair miniature swine with spontaneously regressing melanoma. Their levels increased with the age of the pigs, usually preceding or appearing together with tumor regression and depigmentation. This suggests an antibody-mediated immune response directed against common antigens presented by both malignant and normal swine pigmented cells [155]. Findings in melanoma cells derived from spontaneously regressing Sinclair melanomas suggested that spontaneous regression is associated with higher sensitivity of the melanoma cells to apoptosis [156], the loss of telomerase activity, reduction of telomeric repeats, extensive DNA fragmentation, and formation of apoptotic bodies [157]. Since 1994, the Sinclair miniature swine is produced for research purposes by Sinclair Bio-Resources (Auxvasse, Missouri, USA) as a spontaneously regressing pig melanoma model.

Inheritance of melanoma in the Sinclair miniature swine was intensively studied. However, exact genetic determinants responsible for its development remain to be discovered. A two-locus model was suggested for expression of the exophytic form of melanoma on the basis of complex segregation analysis. One locus lies within the swine major histocompatibility (SLA) complex. The other, yet unidentified, putative dominant tumor-initiator locus segregates independently of the SLA complex. The melanoma-producing allele at this locus is inherited in the heterozygous state and requires a somatic mutation of the normal allele to initiate melanoma development. SLA haplotype B was associated with the expression of Sinclair melanoma. A single dose of the B haplotype is required for full penetrance of the dominant allele at the tumor-initiator locus [158,159,160]. Cytogenetic analyses of three melanoma cell lines from the Sinclair miniature swine revealed specific common chromosomal abnormalities. Structural alteration in chromosomes 2, 3, 6, 7, and 12 were found that probably represent the initial step of melanoma development. In addition, monosomies of chromosomes 2, 4, 7, 10, and 17 and three marker chromosomes (labeled M1, M2, and M3) resulting from chromosomal translocations were detected [161].

### 4.2. Munich Miniature Swine Troll

The Munich miniature swine Troll is historically the second swine model with hereditary melanoma. Literature data about this model and its experimental utilization are very limited. It was established at the University of Munich, Germany, in 1986. One melanoma-bearing boar and two unaffected sows were founders of this herd. They were derived from the herd originally developed from the Hanford and the Columbian miniature swine at the Medical Service Munich. Selective breeding of melanoma-affected animals increased the incidence of malignant tumors to 70%. Benign melanocytic lesions were also observed in addition to melanomas in darkly pigmented (black and red) animals. Skin lesions were already present at birth or they mostly developed within the first two months of life. Complete spontaneous regression of melanomas accompanied by hair and skin depigmentation was also observed in the Munich miniature swine Troll; however, the frequency of regressing pigs was not given. Breeding of Munich miniature swine Troll (manifesting cutaneous melanomas) with the German Landrace (white color, without any skin lesions) and analyses of F1-, F2-, and B1-generations showed that the dominant allele I at the I-locus (responsible for white phenotype) suppressed melanoma lesions. This is explained by a mutation of the *KIT* gene, leading to a failure of melanoblast migration and subsequent lack of melanocytes in the skin of white pigs. The segregation data for skin melanomas in this breed are best explained by a three-locus model with two recessive alleles per locus. An influence of SLA haplotypes on the penetrance of melanocytic lesions was not observed in the Munich miniature swine Troll [162,163]. An in vitro study with melanoma cells of Munich miniature swine Troll suggested a low importance (if any) of blood natural killer (NK) cells for spontaneous regression of melanoma in this animal model [164]. Elevated expression of porcine endogenous retroviruses was detected in melanomas and cell cultures derived from pulmonary metastasis in this swine melanoma model [165]. A similar observation of human endogenous retrovirus K was also reported for human melanomas [166]. Endogenous retroviruses can support cancer formation by inducing chromosomal translocations in somatic cells and promoting immunosuppressor pathways [167]. The publication of Dieckhoff et al. in 2007 [165] is the latest that can be found through PubMed about melanoma research on the Munich miniature swine Troll. Thus, it is not clear if this animal melanoma model still exists.

## 5. The Melanoma-Bearing Libechov Minipig 

### 5.1. Development of the MeLiM Model

Pigs were kept in the Institute of Animal Physiology and Genetics (IAPG) of the Czech Academy of Sciences in Libechov originally for the study of blood groups since 1966. Firstly, two boars and two sows of the Goettingen miniature swine from the University of Goettingen (Institute of Animal Breeding and Genetics, Germany) were imported in December 1966 and another two sows of the same strain in August 1967. The Minnesota miniature pigs from the Hormel Foundation (Austin, USA) and Vietnamese pigs from German zoos were used as foundation stock for development of the Goettingen miniature swine [168]. Then, two imports of the Minnesota miniature pigs (Hormel Foundation, Austin, USA) followed, consisting of two boars and three sows in September 1967 and two boars in February 1969. To maximize genetic variability for the analysis of a wide range of pig blood groups, animals of these two strains of miniature pigs were crossed with pigs of several commercial meat breeds (Canadian Landrace, Cornwall, and Large White) and with Vietnamese pigs. The first few black piglets with cutaneous melanomas were observed in this genetically highly heterogeneous pig population in 1989. They came from mating of two boars (brothers) with four related sows, all without any cutaneous lesions. Selective breeding of melanoma-bearing animals for several generations confirmed genetic predisposition to melanoma with its incidence around 50%. This new pig melanoma model was designated by the acronym MeLiM (melanoma-bearing Libechov minipig; originally melanoblastoma-bearing Libechov minipig) [169,170]. Long-term monitoring of the MeLiM strain showed that values of melanoma incidence varied during individual years depending on tumor burden of parents. For this reason, more affected parental pigs were included in the breeding program, thus increasing melanoma incidence in the MeLiM roughly to 80% in 2018. Tumor devitalization (ischemization) was successfully applied in very affected pigs (see Section 5.8) to increase survival and allow their use in breeding. Currently, eight sows and four boars of the MeLiM line are bred to produce piglets used in experiments. 

Extensive cooperation was established between IAPG (Laboratory of Tumor Biology (LTB)) and other research institutions in the Czech Republic (Czech University of Life Sciences Prague; First Faculty of Medicine of the Charles University Prague; Institute of Microbiology and Institute of Molecular Genetics of the Czech Academy of Sciences, Prague; University of Veterinary and Pharmaceutical Sciences, Brno) for characterization of the MeLiM model. The study of melanoma inheritance in the MeLiM strain was carried out in international cooperation with the INRA/CEA (Institute National de la Recherche Agronomic/Commissariat à l’Energie Atomique, Laboratoire de Radiobiologieet Etude du Génome (LREG), Jouy en Josas, France). Repeated exports of MeLiM animals (melanoma-bearing and melanoma-free) of both sexes and various ages were made from LTB to LREG. They included two boars (age one year) with two pregnant sows (age three years) in June 1997, four boars with six sows (all five months old) in October 1998, six sows (age 6–12 months) in November 2002, and three boars with four sows (age 14–18 months) in June 2008. To reveal genes responsible for melanoma susceptibility in the MeLiM strain, the transported animals were crossed with healthy Duroc pigs in LREG. It is not clear whether the offspring of transported pigs at LREG are currently maintained as a pure MeLiM strain or only as MeLiM × Duroc hybrids. Thus, results obtained in the original MeLiM strain kept in IAPG Libechov and in the MeLiM strain derived from the pigs transported into INRA (Jouy en Josas) may differ.

### 5.2. Histopathological, Biochemical, and Immunohistochemical Characterization

**Figure 2:**
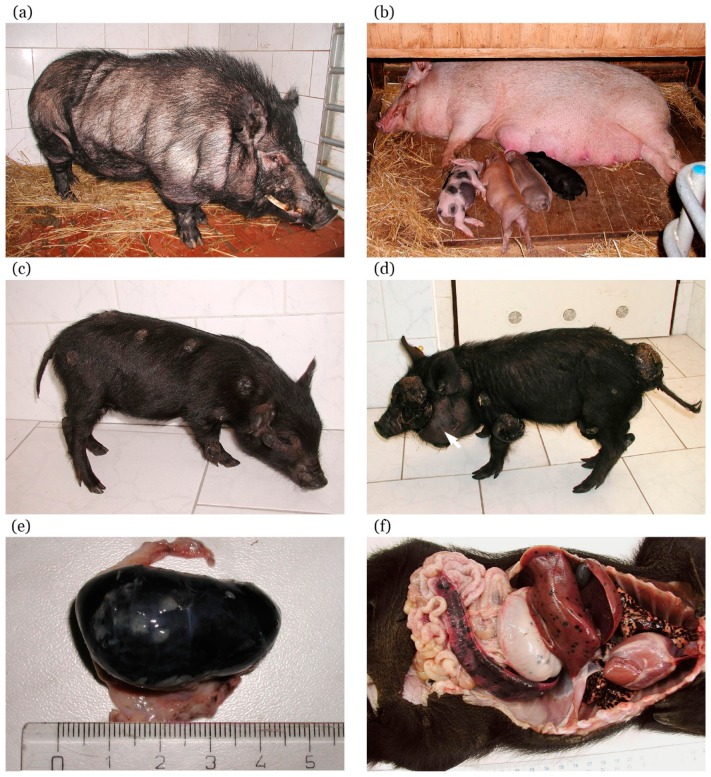
Melanoma-bearing Libechov minipig (MeLiM) swine model of hereditary melanoma: (**a**) black boar of the MeLiM strain after spontaneous regression of melanoma (without any changes in pigmentation) (age three years); (**b**) originally black sow of the MeLiM strain (age four years) after spontaneous regression of melanoma (with almost total depigmentation), together with piglets of different coat color (age three weeks); (**c**) MeLiM piglet with multiple cutaneous nodular melanomas (age six weeks); (**d**) MeLiM piglet showing several large nodular melanomas with local necrosis and beginning cachexia (age seven weeks). Note the vastly increased cervical lymph node (arrow) due to melanoma metastasis; (**e**) very enlarged inguinal lymph node totally infiltrated by metastatic melanoma cells (taken from MeLiM piglet with melanoma progression, age six weeks), scale in cm; (**f**) autopsy of MeLiM piglet that died from melanoma progression (age four weeks). Numerous melanoma metastases (seen as black spots) in visceral organs (lungs, liver, stomach, and spleen) clearly document the malignant characteristic of melanoma in the MeLiM model.

Variability in color coat is observed in the MeLiM animals that reflects the multi-hybrid characteristics of this strain. Pigs are usually black (Figure 2a); however, rusty-red, brown, or white (with black spots) individuals are also rarely found (Figure 2b). Small white spots can infrequently appear in colored animals. Black pigs are the most affected by melanoma. Cutaneous tumors are usually multiple, of deep-black pigmentation, nodular type (with local necrosis in larger tumors), and they are distributed on all body parts (Figure 2c). Rusty-red and brown animals show only one or a few cutaneous melanomas, and white pigs with black spots are without skin lesions. Nevi and superficial spreading melanomas also appear in affected pigs.

Similarly as in the Sinclair miniature swine, skin lesions are found already at birth or they develop shortly thereafter during the first two months of postnatal life. They grow exophytically, reaching sizes of about 15–70 mm, exceptionally up to 150 mm (Figure 2d). Histological observation of cutaneous nodular melanomas revealed variable concentration of brown–black melanoma cells. In the dermis, they formed areas with compact aggregation or were dispersed showing vertical spreading from the basal layer of epidermis into a deeper layer of the dermis (stratum papillare and stratum reticulare) and invading the hypodermis. Thus, these tumors correspond to Clark’s level V of human melanoma. The epidermis was considerably reduced or totally destroyed [169,171]. The malignant characteristic of melanoma in the MeLiM strain is confirmed by presence of numerous metastases. They are commonly found in the lymph nodes (Figure 2e), lungs, and spleen (Figure 2f). Heavily affected animals also demonstrate metastases in other visceral organs such as the stomach, liver, small and large intestine, pancreas, kidneys, heart, and thymus [169,172,173,174]. 

The presence of tyrosinase messenger RNA (mRNA) in the blood is assumed to indicate melanoma metastases [175]. While tyrosinase mRNA was detected by RT-PCR in the blood of MeLiM animals with advanced disease [176], how much this represents the presence of migrating cells contributing to metastasis formation is still unclear. In addition to RT-PCR for the detection of selected pigmented-cell specific mRNAs, novel and more specific techniques are currently being developed for the detection of circulating melanoma cells, applicable for human disease staging, diagnosis, and prognosis [177,178].

Basic biochemical and ultrastructural characterization of the MeLiM melanoma was performed by Borovanský et al. [179]. A very high concentration of melanosomes with a high proportion of melanin (almost 40% of the organelle dry weight) corresponds to deep-black pigmentation of the tumor. Aberrant forms of melanosomes were found by electron microscopy similarly as in the Sinclair miniature swine [180] and human nodular melanoma [181]. Three main melanosome enzymes involved in melanogenesis, biochemical melanoma differentiation, and metastatic activity, i.e., tyrosinase, α-mannosidase, and γ-glutamyltransferase [182,183,184], were detected in the MeLiM melanoma tissue [179].

Immunohistochemical analyses showed further similarities of the MeLiM melanoma with the human one. High expression of *RACK1* was observed in the cytoplasm of cutaneous and metastatic pig melanoma cells. These tumor cells showed also nuclear staining for MITF, a specific marker of the melanocytic lineage. Because of similar findings in human cutaneous melanomas and melanoma metastases, *RACK1* expression could serve as a potential marker of malignancy of human melanoma [185]. Expression of the S100 protein, used for human melanoma diagnosis [186], was also found in cryosections of progressing MeLiM melanomas and cells derived from them in vitro (V. Horak, unpublished observation). Four extracellular matrix proteins, collagen IV, laminin [187], tenascin C, and fibronectin [188,189], as well as matrix metalloproteinase 2 (the enzyme degrading the extracellular matrix) [189], were immunohistochemically found in extracellular spaces of cutaneous melanomas, suggesting their production by the MeLiM melanoma cells. All these proteins can support tumor cell proliferation, migration, and metastases [190,191,192,193]. More than a three-fold increase of tenascin C mRNA in MeLiM melanoma tissue compared to contralateral normal skin was observed, accompanied by elevated protein level [188]. Tenascin C is highly upregulated during wound healing, accompanied by rapid angiogenesis, fibroblast migration to the damaged area, and re-epithelialization by migrating keratinocytes. Elevated tenascin C level is also frequently found in human melanomas, where this protein supports malignant melanocyte survival, invasion, and metastasis [194].

A new computer-supported method for spatial mapping of various metals in tissue sections was developed recently using MeLiM melanomas as a suitable cancer model [195]. The method is based on image registration of digital data obtained from scans of two neighboring cryosections, of which the first one is processed by standard histological staining and the second one is analyzed for metallic content by laser ablation inductively coupled plasma mass spectrometry (LA-ICP-MS). Detailed histological analysis of cutaneous melanomas sampled from MeLiM pigs aged 4–22 weeks revealed four structurally different tissue zones—growing melanoma tissue (GMT), early spontaneous regression (ESR), late spontaneous regression (LSR), and fibrous tissue (FT)—whose presence, size, and proportion in melanoma tissue changed with animal age and advancing melanoma regression. This pilot study showed the highest concentrations of zinc and cooper in growing melanoma tissue, whereas the lowest ones were found in fibrous tissue. Both these metals are important players in various cancer diseases. Zinc level is increased in the majority of human melanomas but copper level is elevated only in some of them [196]. Application of matrix-assisted laser desorption/ionization mass spectrometry imaging (MALDI MSI) revealed four ion peaks, *m/z* 3044, 6011, 6140, and 10180, which were overexpressed in MeLiM melanoma tissue in comparison to healthy skin. Moreover, the ion peaks at *m/z* 6011 and 6140 were overexpressed in the GMT region. These findings agree with the high zinc content observed in this region in a previous study, leading to the assumption that both peaks represent metallothioneins [197]. Elevated metallothionein content in the MeLiM melanoma was already detected previously by the adsorptive transfer stripping differential pulse voltammetry Brdicka reaction [198]. Overexpression of metallothioneins was associated with a poor prognosis in human cutaneous melanoma [199]. These recent studies of MeLiM melanoma show the usefulness of this swine model for basic melanoma research and suggest possibilities for its further use in the search for markers of melanoma progression and spontaneous regression that could serve in clinical practice.

### 5.3. MeLiM Melanoma Progression and Spontaneous Regression

In the MeLiM model, multiple cutaneous melanomas found on various parts of body develop differently over time for each individual. Two main situations may occur—cancer progression and/or spontaneous regression [200]. Small cutaneous tumors (found at birth or developed shortly thereafter) initially grow in all affected piglets.

**Figure 3:**
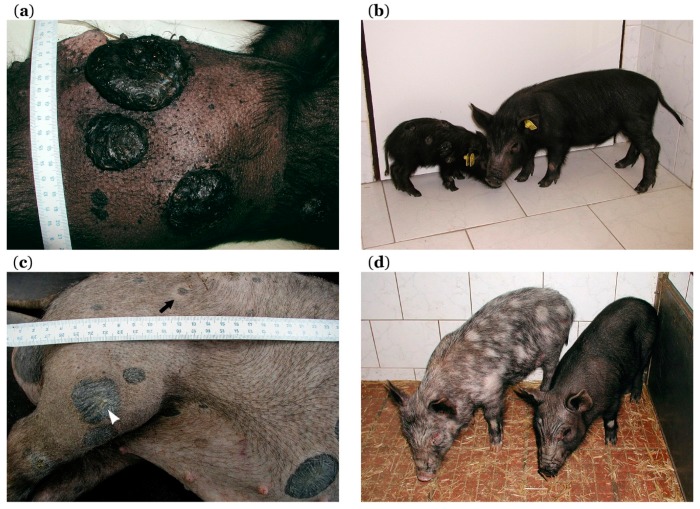
Progression and spontaneous regression in MeLiM model: (**a**) three growing cutaneous nodular melanomas (with local necrosis) are well visible after shaving off the bristles (age 11 weeks); (**b**) comparison of two MeLiM siblings, one with melanoma progression causing heavy cachexia and body size reduction (left side) and one with spontaneous regression and normal body size (right side) (age 10 weeks); (**c**) flattening and graying of originally nodular melanoma (arrowhead) and halo around one smaller melanoma (arrow), together with partial depigmentation of skin and bristles observed in MeLiM pig with ongoing spontaneous regression of melanoma (age four months); (**d**) partial bristle and skin depigmentation versus preserved black pigmentation in two MeLiM siblings with spontaneous regression of melanoma (age 5.5 months); scales in cm.

In a smaller part of affected piglets (about 5–30% depending on disease burden in parents), cancer progression continues. Melanoma progression mainly affects black piglets, while it is very rare in rusty-red and brown ones. Cutaneous melanomas grow further reaching a large size (Figure 3a), with occasional bleeding and local necrosis. These heavily affected piglets initially lag in bodyweight gains behind their less affected (spontaneous regression showing) siblings (Figure 3b). At the later stage, they lose weight and develop strong cachexia with melanoma progression. Extensive metastases are observed in the lungs, lymph nodes, and spleen. Metastases in lymph nodes, mainly in cervical and inguinal areas, are also macroscopically visible in some animals due to their increasing size (Figure 2d, arrow). Additionally, metastases are present in the liver, various parts of the gastrointestinal system (Figure 2f), thymus, heart, and brain [169,173]. The animals with progressive melanoma usually die during the first three months of age. The main cause of death seems to be breathing difficulties and insufficient oxygen supply of the whole organism due to severe damage of lung tissue with a vast number of melanoma metastases.

Spontaneous regression of melanoma is observed in the majority of MeLiM piglets. After the initial period of growth, tumors begin to flatten, reduce in size, and change color from black to gray. Piglet body weight reaches normal or almost normal values. Melanoma regression is usually associated with skin and bristle depigmentation (Figure 3c, white arrowhead). It starts as sparsely dispersed white bristles over the body or localized discoloration around several cutaneous tumors. A halo effect around some melanomas is also observed (Figure 3c, black arrow). Then, depigmentation gradually extends to the surrounding parts of the body. This depigmentation spreads sometimes to almost the entire body leading to the originally black pig becoming nearly white (Figure 2b) [169,171]. A specific CD4 haplotype was observed in T-lymphocytes to be related to the depigmentation during regression [201]. However, the black pigmentation is rarely maintained in MeLiM pigs with spontaneous regression (Figure 3d). Skin depigmentation was also observed in melanoma patients with spontaneous regression and/or treated by immunotherapy [72]. These color changes of the skin suggest the activation of immune cells against an antigen that is common to melanoma cells and normal melanocytes.

The spontaneous regression is a very dynamic process in which melanoma cells are gradually destroyed and tumor tissue is replaced with the fibrous tissue. Vincent-Naulleau et al. monitored spontaneous regression of melanoma in a colony of MeLiM pigs that was derived from the MeLiM animals transported from the Czech Republic to France and in their Duroc crossbreeds. They observed that the time course of spontaneous regression was dependent on tumor growth. In fast-growing tumors, spontaneous regression appeared between the third and fourth month, whereas slow-growing tumors demonstrated it between the fifth and seventh month. Moreover, two regression phases were observed in some exophytic tumors that were present at birth. The early regression (between the second and the third month) was followed by a transitional period of relapse and tumor growth (between the 2.5th and 4.5th month) and finally with the latest regression phase (between the 3.5th and sixth month) [171]. Our time-lapse immunohistochemical study of exophytic melanomas taken from pigs of the original MeLiM strain (from three weeks to eight months of age) showed only one regression phase. Expressions of fibronectin, tenascin C, collagen IV, laminin, and MMP2 increased up to the 10th week of age. In older animals, gradual destruction of melanoma cells and rebuilding of melanoma tissue into the fibrous tissue was observed. In agreement with this process, the expression of collagen IV, laminin, and MMP2 declined, whereas the expression of fibronectin and tenascin C raised in the arising fibrous tissue. The age of 10 weeks seems be a turning point in the transition between the initial melanoma growth phase and subsequent spontaneous regression phase [187,189].

Spontaneous regression does not occur synchronously in all melanoma sites on the body. Its duration depends on the number and size of melanoma deposits. The whole process of spontaneous regression is usually completed around 6–12 months of age.

### 5.4. Genetic Findings

The development of melanoma in pigs is a polygenic process [202]. The *CDKN2A* locus causative in human familial melanoma was studied in MeLiM pigs; however, haplotype analysis, allelic association, and linkage analysis led to exclusion of this gene from candidates for melanoma susceptibility [203]. Later experiments revealed that MeLiM melanoma is inherited as an autosomal dominant trait with incomplete penetrance. The inheritance of melanoma was seen preferably in black animals. Association of regions harboring *CDK4* and *BRAF* genes was not found; however, another three candidate regions which correspond to human regions with melanoma candidate loci were observed [204]. For the black coat color, a variant allele of the *MC1R* gene was found (marked as MC1R*2) to be associated with melanoma development. This is in agreement with the fact that human variant alleles of *MC1R* may increase melanoma risk independently of UV exposure [202]. Comparative expression analysis revealed that the *RACK1* gene is overexpressed in melanoma metastases compared to normal melanocytes. This finding is consistent with results observed in human melanoma patients [185]. Functional studies highlighted that the *MITF* gene has potential involvement in porcine melanoma biology; however, direct association of this gene with melanoma development was not confirmed [205]. A 450-kb duplication in the *KIT* gene was found to be responsible for white or belt coat color in pig, as it prevents migration of embryonic melanoblasts to skin. Diverse *KIT* mutations were found in various human cancers, including melanoma, and one variant showed a significant association with cutaneous invasion, melanoma development, and tumor ulceration in the MeLiM strain [206]. 

Genome-wide time-dependent profiling was conducted to analyze molecular mechanisms involved in MeLiM spontaneous melanoma regression. Among other results, downregulation of genes involved in cell cycle and DNA replication, recombination, and repair was observed in tumors at the 28th, 49th, and 70th day of age in a piglet with spontaneous regression, suggesting the reduced proliferative capacity of melanoma cells. Moreover, upregulation of monocyte/macrophage-related genes at the same time points was accompanied by tumor-infiltrating macrophage infiltration observed in tumor histological sections. At three months of age, upregulation of different T-cell receptor (TCR) chains, as well as T-cell-associated cytokines, together with dramatic downregulation of genes involved in melanogenesis, confirms T-cell activation and loss of melanoma cells at the later phases of regression [207]. In addition, suppression subtractive hybridization was used to study gene expression in progressive and regressive MeLiM melanoma tissue. Verification by RT-PCR and immunohistochemistry confirmed upregulation of CD9 and retinoic acid responder 1 gene (*RARRES1*) in regressive tumors, while *MITF* was upregulated in progressive melanomas [208].

A genome-wide association study performed on 190 animals of the MeLiM × Duroc pedigree revealed several loci on chromosomes 2, 5, 7, 8, and 16, showing significant associations with melanoma occurrence and progression (i.e., clinical ulceration and presence of metastasis). The most significant region associated with melanoma occurrence was located on chromosome 5 harboring the *NUAK1* gene encoding AMP-activated protein kinase (AMPK)-related protein kinase 5 (ARK5) [209]. ARK5 is known to promote survival and invasion of cancer cells and is probably activated by the Akt kinase [210]. GWAS analysis of tumor ulceration revealed a region on chromosome 16 nearby the *IRX4* gene (iroquois homeobox gene) [209], previously identified as a risk factor in human prostate cancer [211]. Interestingly, *IRX4* is located only 600 kb from the *TERT* gene. Mutations in *TERT* promoter are associated with both familial and sporadic melanoma [40]. Genes associated with metastasis in MeLiM were identified on chromosomes 2 (coding long non-coding RNAs (lncRNAs) with functions in tumor suppression and metastasis formation) and on chromosome 8, harboring the *HERC3* (probable E3 ubiquitin–protein ligase) gene [209]. HERC3 is an endosomal protein with probable ubiquitin–protein ligase function. *HERC3* mutations were observed in gastric and colorectal cancers [212]. In MeLiM melanoma, an additional 12 loci, previously reported to associate with melanoma in human, were identified. Several novel gene candidates associated with MeLiM melanoma, not yet reported in human, were also revealed [209].

MicroRNAs (miRNAs) are in the center of current research because they play important roles in all processes in the cell, and they also participate in melanoma development [213]. Analysis of miRNA in MeLiM tumors revealed significant upregulation of let-7b, miR-193b, miR-21, miR-221, and miR-222 in regressive tumors in contrast to miR-92a, which was upregulated in progressive tumors. The expression of miR-92a, let-7b, and miR-193b in regressive MeLiM tumors was in contrast to previous findings in progressive human tumors, suggesting that such miRNAs could be potential actors in the regression process in MeLiM cutaneous melanoma. MiR-193b could regulate cell-cycle-related genes during regression of cutaneous melanoma [214].

### 5.5. Hematological Findings

Hematological monitoring is an integral part of the diagnosis of cancer and of the subsequent treatment. Values of various hematological parameters, such as leukocyte and neutrophil counts and their ratios (neutrophil–lymphocyte and platelet–lymphocyte ratios), can be used as prognostic markers in different types of cancer [215,216,217,218] including melanoma [219,220]. Elevated leukocyte count with neutrophilia was found in metastatic melanoma patients [221]. A baseline neutrophil–lymphocyte ratio lower than five was associated with improved survival of metastatic melanoma patients treated with ipilimumab [222]. Another study of patients with early-stage (I–III) melanoma showed worse survival with a baseline neutrophil–lymphocyte ratio lower than 2.5 [223]. Thrombocytosis [224] and low concentration of blood hemoglobin [225] predicted metastatic disease and worse survival in melanoma patients. Anemia is commonly found in cancer patients indicating a poor prognosis. It is a multifactorial process that is often connected with iron deficiency as a major causal factor [226], manifesting as decreased erythrocyte count and lower hematocrit. The level of blood iron and iron homeostasis is important for both innate and adaptive immunity response [227,228]. One of the many important functions of iron is the regulation of immune cell distribution [229].

Hematological analyses are also important for monitoring animal cancer models, as shown in our recent study [200]. Basic hematological parameters of MeLiM animals with melanoma progression or spontaneous regression were compared to healthy (white, melanoma-free) animals from 5–18 weeks of age. Iron deficiency and microcytic hypochromic anemia were observed in all MeLiM pigs. The group of pigs with melanoma progression was characterized by the lowest values of red blood cell count, hematocrit, and concentration of hemoglobin, as well as by the highest number of platelets. Moreover, a very high number of neutrophils was found (measuring differential white blood cell counts), driving the high number of white blood cells observed in these animals. In the spontaneous regression group, higher values of red blood cell count, hematocrit, and concentration of hemoglobin, together with a lower number of platelets, were ascertained. Thus, monitoring hematological parameters enables distinguishing (together with macroscopic, histologic, immunological, and immunohistochemical observations) MeLiM piglets with progression and spontaneous regression in early postnatal development. These findings extend the characterization of the MeLiM model and show its further similarities with melanoma patients.

### 5.6. Immunological Findings

Immune cells infiltrating tumors, including melanoma, are responsible for anti-tumor immunological surveillance. However, some tumor-associated immune cell types (such as macrophages and neutrophils) can also support cancer progression depending on tumor milieu [230,231]. A higher infiltration of cutaneous melanomas with lymphocytes is associated with better prognosis and longer survival of melanoma patients. The cytotoxic CD8^+^ T-lymphocytes collaborating with the CD4^+^ T-helper cells were found to be the most important components [232,233]. Promising results of treatment of metastatic melanoma patients with adoptive transfer of tumor-infiltrating lymphocytes (TILs) confirmed their anti-cancer effectiveness [234].

The MeLiM animals with melanoma spontaneous regression represent a promising immunological model for monitoring immune cells participating in anti-melanoma reaction. Flow cytometry revealed two DP T-lymphocyte subpopulations, i.e., melanoma-associated CD4^+^/CD8^high^ T-lymphocytes in peripheral blood and CD4^+^/CD8^high^ TILs in melanoma tissue (together with CD4^−^/CD8^+^ T-lymphocytes), which expanded during melanoma regression. They showed a similar expression of selected CD markers between different pigs and different melanoma loci among the same pig, suggesting that they are effector/memory αβ T-cells considerably involved in spontaneous regression of MeLiM melanoma [235]. It is important to mention, that CD4^+^/CD8^+^ cells are more frequent in pigs, reaching up to 60% of total T-cell counts in adult pig blood, in contrast to 3% in human [236]. The number of DP T-cells naturally increases during the life of pigs [237], which may mask the increase caused by MeLiM regression. Nonetheless, MeLiM peripheral DP cells differ in the intensity of CD8 expression, with CD8^high^ expression in the melanoma-bearing animals in later stages of tumor regression compared to CD8^low^ positivity in their melanoma-free littermates (both groups at the age of eight months). Importantly, a unique DP cell subpopulation was identified in the blood of regressive MeLiM animals, representing one T-cell clone carrying a mono-specific TCRβ receptor, which is supposed to be responsible for melanoma regression [235]. Our unpublished data about cytokine production of DP T-cells suggest that these cells represent a non-naïve (activated, recirculating) lymphocyte subpopulation with immunomodulatory activity. Compared to single-positive T-cell populations, where 30% and 50% of CD4 single-positive and cytotoxic T-cells produced IFNγ and/or tumor necrosis factor α (TNFα), respectively, almost 60% of DP T-cells were cytokine producers.

Although the significance of CD4^+^/CD8^+^ DP T-cells in cancer conditions remains unclear, they are mentioned to play an important role at peripheral sites. Their functions are probably the consequence of various microenvironments found across different types of tumors. Anti-tumor actions of DP cells were described in various tumor types [236]. Bagot et al. isolated a clone of DP cells with a CD4^+^/CD8^+^ dim phenotype from the cutaneous infiltrate of a patient with T-cell lymphoma. These cells were major histocompatibility complex class I (MHC I) restricted and cytolytic against autologous tumor cells in vitro [238]. Concerning clinical outcomes, De Marchi et al. described the presence of CD4^+^/CD8^+^ T-cells in cutaneous lesions in mycosis fungoides. Their presence was associated with a slightly slower progression of the disease [239]. A significant increase of DP cells was also noted in human malignant melanomas and their metastases. Increased numbers of DP cells were observed in about 60% of melanomas compared to peripheral blood. A high proportion of these cells were TNF-α-producing in response to autologous melanoma cells. They were also characterized by higher secretion of IL-13, IL-4, and IL-5 compared to single-positive cells [240].

### 5.7. Skin Microbiome

Microbiome is a term for the community of microorganisms (bacteria, archaea, fungi, protozoa, viruses) living at a given environment, e.g., on the epithelial surfaces of the mammalian body. The local microbiome affects functions of the epithelial barrier and regulates immunity [241]. In cancer, microorganisms may directly contribute to cancer development (e.g., in gastric, colorectal, cervical, and hepatocellular cancer or lymphoma) and may modify patients’ immunity and response to therapy [242]. The gut microbiome is increasingly recognized as a modulator of response to anti-cancer treatment, particularly to immune checkpoint inhibitors [242,243,244]. The skin microbiome is much less explored. In human, the skin microbiome was analyzed in a search for a diagnostic tool for melanoma and melanocytic nevi. However, no significant differences between melanoma and nevi microbiomes were found [245].

In MeLiM piglets, the possible involvement of skin microbiome in melanoma development was studied. Melanoma surface and healthy skin (5 cm from the melanoma lesion) were compared by matrix-assisted laser desorption/ionization time of flight mass spectrometry (MALDI-TOF MS) of cultured microorganisms [246], as well as 16S ribosomal RNA (rRNA) analysis [247]. Using MALDI-TOF, a clear significant difference between the proportions of bacteria on healthy skin and melanoma was observed, with *Staphylococcus sciuri*, *Lactococcus lactis*, and *Staphylococcus cohnii* being typical for healthy skin, while *Staphylococcus chromogenes*, *Staphylococcus hyicus*, and *Enterococcus faecalis* were abundant on the melanoma surface [246]. To monitor the possible involvement of skin microorganisms in melanoma development, skin and melanoma scrapes were analyzed by 16S rRNA PCR and denaturing gradient gel electrophoresis (PCR-DGGE) in six-, eight-, 10-, and 12-week-old MeLiM piglets, which is the age when the regressive/progressive phenotype develops. Similarly to MALDI-TOF results, the predominance and distribution of bacterial genera were different between skin and melanoma samples. The melanoma surface microbiome showed significantly higher microbial diversity than healthy skin, which might be partially caused by melanoma ulceration. The number of *Fusobacteria* was higher in melanoma samples compared to healthy skin and also in progressing melanomas compared to regressing ones. In addition, the quantity of *Fusobacterium necrophorum* increased with the age of piglets with progressing melanoma [247]. In human, the abundance of *Fusobacterium* (particularly *F. nucleatum*) in the gut is connected with colorectal cancer development and progression [248,249,250]. Additional studies of the MeLiM model are needed to elucidate the possible effects of the skin microbiome on melanoma development or immune reactions in the skin.

### 5.8. Experimental Therapy of MeLiM Melanoma by Tumor Devitalization

Tumor devitalization (also called devascularization) was developed by the Czech surgeon Karel Fortýn (1930–2001) and suggested as a surgical operation technique for treatment of solid tumors. The principle of this technique is total closure of blood supply (ischemization) to tissue by ligating all vessels—arteries and veins—with non-absorbable material and leaving the treated tissue in situ. This procedure was firstly experimentally tested in healthy (tumor-free) miniature pigs held in IAPG. Segments of the small or large intestine were devitalized by ligation of the mesenteric arteries and veins. Both ends of the devitalized intestine were also ligated (forming a blind loop), left in situ together with its content, and the intestinal passage was renewed by anastomosis. The experimental minipigs survived without any health complications, and the isolated intestinal segments were gradually destroyed over four weeks without causing sepsis [251]. Based on these promising results, devitalization was successfully applied in several elder patients (age 57–82 years) with inoperable colorectal carcinoma. Revision operations showed a small fibrous residue at the site of the original tumor only, and visceral metastases, ascertained before devitalization, were not found. No cancer recurrence was observed in the patients. They died 4–7 years later of a heart attack or stroke [252]. Recently, another case report of a patient with invasive metastatic colorectal carcinoma who survived more than 14 years after devitalization, with no sign of malignancy revealed on computed tomography (CT) scans at present, was published [253]. Using healthy minipigs in IAPG as an anatomical and physiological model similar to human, devitalization of the kidney [254,255], stomach [256], rectum, and sigmoideum [257] was also carried out to acquire practical skills and experimental knowledge as a prerequisite for possible clinical utilization. In all cases, the devitalized tissues were resorbed and no side effects were observed.

Development of the MeLiM strain with hereditary melanoma gave us a very suitable animal model to experimentally test the effects of tumor devitalization in vivo. Devitalization of cutaneous melanoma is a relatively simple surgical technique. Partially overlapping mattress stitches are conducted around the tumor base and strongly tightened; then, the tumor is left in situ without any excision [174]. More than 40 MeLiM animals of both sexes (age 1–2 months) with progressively growing multiple cutaneous nodular melanomas and metastases in inner organs (lymph nodes, spleen, and liver) were used in the first larger study. Devitalization of single cutaneous melanoma led to a gradual melanoma cell destruction in all other non-treated cutaneous melanomas, as well as inner organ metastases, over 4–6 months. Neither side effects (with the exception of local or generalized depigmentation) nor any health complications were ascertained [169]. Melanoma cell destruction was also well documented biochemically, showing a great reduction in α-mannosidase and tyrosinase activities in non-treated melanomas taken six months after devitalization of another cutaneous melanoma [179].

Increased expression of two heat-shock proteins (HSPs)—HSP70 and gp96—was demonstrated immunohistochemically and by Western blotting in the devitalized melanoma as early as one day after treatment, which persisted for the next two weeks. The growing proportion of tumor-infiltrating lymphocytes (cytotoxic T-lymphocytes and DP T-lymphocytes) was proven thereafter by flow cytometry in non-treated cutaneous melanomas [258]. Both monitored HSPs are able to form complexes with immunogenic peptides derived from cancer cells and, through antigen-presenting cells, they activate cytotoxic T-lymphocyte responses against the HSP-bound peptides [259,260,261]. Based on these findings, HSP70 and gp96-peptide vaccines derived from autologous tumor lysate were tested as a novel promising approach for the treatment of various malignancies including metastatic melanoma. Vitespen (formerly Oncophage) was the first personalized gp96-peptide cancer vaccine developed by the Antigenics Inc. (New York, NY, USA) and used in randomized clinical trials [262,263,264,265,266]. Our finding from devitalization experiments in the MeLiM model are in accordance with this therapeutic trend. Long-term overexpression of HSPs, followed by significant tumor lymphocyte infiltration, suggests that melanoma devitalization in the MeLiM model elicits a cell-mediated anti-tumor immune response. Thus, devitalization can be considered as an immunotherapeutic technique (auto-vaccination by necrotic tumor tissue from devitalized melanoma). At present, we apply melanoma devitalization for therapy of the MeLiM pigs with progressing melanoma to prolong survival and allow their inclusion as parental animals in the MeLiM herd. Their utilization in breeding schemes increases the incidence and severity of melanoma in this animal model.

## 6. Concluding Remarks

Enormous work was done in melanoma research, and even more remains to be elucidated. The study of intrinsic tumors and in vitro cultured cells, as well as the employment of animal models, enables us to be closer to understanding the disease etiology. The new genetic discoveries may help us to find new therapeutic targets or molecular reporters to monitor the disease development or therapy efficacy. Understanding the role of the immune system in melanoma control is crucial for immunotherapies. 

Animal models are indispensable in melanoma research. Various mouse models are prevailingly utilized; however, swine models seem to be more appropriate due to anatomical, physiological, biochemical, and genetic similarities with human. Using genetic engineering, various transgenic swine models are available for biomedical research [267,268] including cancer [269,270]. However, no transgenic melanoma swine model was developed until now.

Several advantages of pig models highlight their importance in melanoma research. The pig skin structure and melanocyte distribution in pigmented skin more closely resemble the human situation (in contrast to mouse skin). Larger litters enable studying progression and regression by comparing sibling pairs. The long lifespan (12–18 years in miniature pig [271]) enables long-term monitoring of pig breeds and experimental outcomes. Large animal models also allow repeated blood and tissue sampling during the life of the individual to monitor the disease development. For example, repeated sampling in MeLiM model allows us to monitor spontaneous regression course and the involvement of immune cells in the disease control. Outcomes of such studies have the potential to bring new knowledge that would be usable in studies of human melanoma and its treatment. 

Two already established and well-characterized swine models with spontaneous, hereditary melanoma—the Sinclair miniature swine and the melanoma-bearing Libechov minipig—showing many similarities with human melanoma, seem to be the best choice for melanoma study. These models closely resemble each other with respect to melanoma development, its spontaneous regression, and histopathological findings. However, genes responsible for predisposition to melanoma remain to be identified in both strains. The Hormel (Minnesota) miniature pig used in the establishment of the Sinclair and MeLiM models could carry susceptibility genes for melanoma. The Sinclair miniature swine is generally usable as a spontaneously regressing melanoma model because this biological process appears in most animals. The advantage of the MeLiM model is that, in addition to the spontaneous regression of melanoma observed in most animals, melanoma progression causing death is regularly observed in about 5–30% of affected pigs (depending on the disease burden in parenting individuals). Using repeated tissue and blood analyses and monitoring the health status of piglets from birth, we are able to distinguish pigs with spontaneously regressing melanoma from those with progressing melanoma and use them separately for studying the regression phenomenon and for the development of new techniques for melanoma treatment. Cooperation with research groups that are interested in large animal model melanoma research is desirable to maintain this unique swine model.

## Abbreviations

**BAP1**: BRCA1-associated protein-1, **CDK4**: cyclin-dependent kinase 4, **CDKN2A**: cyclin-dependent kinase inhibitor 2A, **CTLA-4**: cytotoxic T-lymphocyte-associated protein 4, **DP**: double-positive, **EGF**: epidermal growth factor, **GWAS**: genome-wide association study, **HER**: human epidermal growth factor receptor, **HSPs**: heat-shock proteins, **IFN**: interferon, **IL**: interleukin, **MALDI-TOF MS**: matrix-assisted laser desorption/ionization time of flight mass spectrometry, **MAPK**: mitogen-activated protein kinase, **MC1R**: melanocortin 1 receptor, **MeLiM**: melanoma-bearing Libechov minipig, **miRNAs**: microRNAs, **MITF**: microphthalmia-associated transcription factor, **NF1**: neurofibromin 1, **PD-1**: programmed cell death protein 1, **PD-L1**: programmed death-ligand 1, **PDX**: patient-derived xenograft, **PI3K**: phosphatidylinositol 3-kinase, **POT1**: protection of telomeres protein 1, **PTEN**: phosphatase and tensin homolog, **RACK1**: receptor for activated C kinase 1, **SCID**: severe combined immunodeficiency, **SLA**: swine leukocyte antigen (swine major histocompatibility complex (MHC)), **TIL**: tumor-infiltrating lymphocytes, **TNF**: tumor necrosis factor, **Xmrk**: *Xiphophorus* melanoma receptor tyrosine-protein kinase.
